# Generation of Five Human Lactoferrin Transgenic Cloned Goats Using Fibroblast Cells and Their Methylation Status of Putative Differential Methylation Regions of *IGF2R* and *H19* Imprinted Genes

**DOI:** 10.1371/journal.pone.0077798

**Published:** 2013-10-30

**Authors:** Li Meng, Yongjie Wan, Yanyan Sun, Yanli Zhang, Ziyu Wang, Yang Song, Feng Wang

**Affiliations:** 1 Jiangsu Livestock Embryo Engineering Laboratory, Nanjing Agricultural University, Nanjing, China; 2 Human and Animal Physiology, Wageningen University, Wageningen, The Netherlands; 3 Animal Breeding and Genomics Centre, Wageningen University, Wageningen, The Netherlands; University of Bonn, Institut of experimental hematology and transfusion medicine, Germany

## Abstract

**Background:**

Somatic cell nuclear transfer (SCNT) is a promising technique to produce transgenic cloned mammalian, including transgenic goats which may produce Human Lactoferrin (hLF). However, success percentage of SCNT is low, because of gestational and neonatal failure of transgenic embryos. According to the studies on cattle and mice, DNA methylation of some imprinted genes, which plays a vital role in the reprogramming of embryo in NT maybe an underlying mechanism.

**Methodology/Principal Findings:**

Fibroblast cells were derived from the ear of a two-month-old goat. The vector expressing *hLF* was constructed and transfected into fibroblasts. G418 selection, EGFP expression, PCR, and cell cycle distribution were applied sequentially to select transgenic cells clones. After NT and embryo transfer, five transgenic cloned goats were obtained from 240 cloned transgenic embryos. These transgenic goats were identified by 8 microsatellites genotyping and southern blot. Of the five transgenic goats, 3 were lived after birth, while 2 were dead during gestation. We compared differential methylation regions (DMR) pattern of two paternally imprinted genes (*H19* and *IGF2R*) of the ear tissues from the lived transgenic goats, dead transgenic goats, and control goats from natural reproduction. Hyper-methylation pattern appeared in cloned aborted goats, while methylation status was relatively normal in cloned lived goats compared with normal goats.

**Conclusions/Significance:**

In this study, we generated five *hLF* transgenic cloned goats by SCNT. This is the first time the DNA methylation of lived and dead transgenic cloned goats was compared. The results demonstrated that the methylation status of DMRs of *H19* and *IGF2R* were different in lived and dead transgenic goats and therefore this may be potentially used to assess the reprogramming status of transgenic cloned goats. Understanding the pattern of gene imprinting may be useful to improve cloning techniques in future.

## Introduction

There are many ways available to produce transgenic animals, such as pronuclear injection and sperm-mediated gene transfer [1]. Somatic cell nuclear transfer (SCNT), which uses preselected genetically modified cells as donor nuclei, is recognized as a more efficient way to produce transgenic animals until now [2]. SCNT is widely used to generate transgenic pigs and cows which have improved meat or milk quality [3], [4]. However, researches about the application of transgenic cloned goats are less. Dairy goats are preferable to other species to produce recombinant proteins in milk for the following reasons [5]. First, dairy goats produce more milk than rabbits and mice. Second, unlike cows, goats have earlier sexual maturation, shorter breeding generation interval, and more offspring per parity. Third, goat milk is safer for human, because there is no severe infectious disease like mad cow disease. Fourth, goats are more amendable to be cloned by NT and the cloned offspring have a longer life span. Human Lactoferrin (hLF) plays important physical roles in anti-bacterial, anti-viral, anti-fungal infections, and cancer prevention [6]. Many previous attempts have been intended to produce hLF for structure function studies [7], [8], but the previous methods were not suitable for very large-scale production. Transgenic goat mammary gland is an alternative way of producing this protein, because of large milk yield and precise posttranslational modifications [9]. Thus, we decided to investigate the effective way of producing *hLF* transgenic cloned dairy goats.

Although SCNT is the preferred approach to produce transgenic goats and other large animals, the success percentage remains low [10]. Early embryonic and fetal loss, stillbirth, postnatal loss, and abnormalities account for this. The reason of low birth survival is probably due to incomplete reprogramming of epigenetic marks in differentiated donor cells [11]. DNA methylation of imprinted genes is an important way to regulate epigenetic reprogramming. Imprinted genes are epigenetically marked with their parental origin and a given parental allele is expressed while the other is repressed [12]. Knockdown or deletion experiments have demonstrated that imprinted genes participate in ongoing development, such as energy metabolism in life cycles [13]. However, the expression level of imprinted genes was mainly affected by the DNA methylation [14]. Aberrant imprinting has been shown to result in a number of diseases, including a variety of developmental syndromes, such as Prader-Willi syndrome and cancers [15]. During embryonic development, the methylation status of some imprinted genes is dynamic and has spatial and temporal requirements [12]. *H19* and Insulin-like growth factor 2 receptor (*IGF2R*) are essential for normal development and therefore the most studied imprinted genes involved in foetal growth regulation. Abnormal DNA methylation of differential methylated regions (DMR) in imprinted genes may cause their biallelic expression or silencing [12].

Many details of DNA methylation of imprinted genes have been elucidated in cloned mice [16] and swine [12], [17]. However, little is known in ruminants, especially for cloned goats, due to lack of genomic sequence [18]. Goat is an economically important agricultural species and animal model for biology studies as well. The interest in elucidating imprinting mechanisms in ruminants has grown in recent years due to the great incidence of abnormal development in animals produced from assisted reproductive technologies such as SCNT. Additionally, along with the more available whole-genome sequences from Goat Genome Project, it is possible to systematically study the event of methylation imprints in goats [18]. A more thorough understanding on how methylation imprints is reprogrammed during cloning and how the methylation is maintained during development will help to improve the technique of NT.

In this study, we produced five transgenic cloned goats successfully using SCNT. Three were born alive and the other 2 were dead during gestation. Furthermore, our study on the DNA methylation in live and dead transgenic cloned goats demonstrated that *H19* and *IGF2R* played significant roles in the nuclei of donor cells reprogramming in NT, which could explain the death of transgenic cloned goats aborted in this study.

## Materials and Methods

### Construction of the *hLF* Mammary Gland-specific Expression Vector

**Figure 1:**
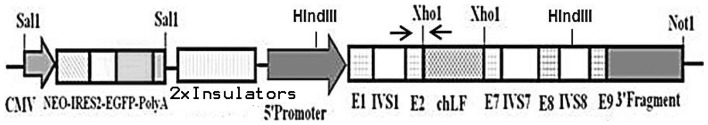
Schematic diagram of the goat β-casein *hLF* transgene vector pBC1-hLF-Neo-IRES2-EGFP. (1) Insulator: chicken β-globin insulator (2×); 5′promoter: goat β-casein promoter; E1, E2, E7, E8, and E9: β-casein exon; IVS1, IVS7, and IVS8: β-casein intron; 3′ fragment: β-casein 3′ genomic fragment. (2) Two fragments containing the hLF gene and Neo-IRES2-EGFP genes were cloned into the Xho I and Not I sites, respectively. Hind III enzyme sites and “→←” present probe position for southern blot.

The *hLF* cDNA (GenBank accession no. X53961) was artificially synthesized and inserted into the vector pBluescript II SK (+) by Invitrogen Service, (Shanghai, China). The vector also contained a signal peptide sequence, a coding area, a stop codon (TAA), and two *XhoI* restriction sites. The *hLF* cDNA sequence was isolated from pBluescript II SK (+) and inserted into the vector PBC1 (Invitrogen, USA) by *XhoI* site. The obtained vector was termed pBC1-hLF. 794 bp coding region of neomycin (*neo*) gene was obtained from pcDNA3.1 vector (Invitrogen, USA) and cloned into pIRES2-EGFP (Clontech, USA). The obtained vector was named pNeo-IRES2-EGFP. After deleting *Sal I* and *Xho I* sites, this vector was used as the template to amplify the fragment of Neo-IRES2-EGFP by PCR, Phusion® High-Fidelity DNA Polymerase (NEB, UK) and the following procedure: pre-denaturing at 98°C for 2 min, and 30 cycles with denaturing for 20 s at 98°C, annealing for 30 s at 61°C, extension for 2 min at 72°C, and a final extension for 8 min at 72°C. After sequencing, this fragment was cloned into the pBC1-hLF vector under the control of the cytomegalovirus promoter. The final vector was named as pBC1-hLF-Neo-IRES2-EGFP. The schematic diagram of this vector was shown in Figure 1. Primers used for the plasmid construction was listed in Table S1 in File S1.

### Inductive Expression of *hLF* Mammary Gland-specific Expression Vector *in vitro*

We isolated the primary mammary epithelial cells (MECs) from a lactating dairy goat 60 days post parturition using the methods described in the previous study [19]. Anti-cytokeratin 18 (Abcam, USA), which is the epithelial cells specific protein, was used to identify this cell by immunofluorescence. The purified passage 4 goat MECs were transfected with the vector pBC1-hLF-Neo-IRES2-EGFP. The G418 resistant MECs were confluent in 24-well plates through 4 weeks of G418 selection. Then cells were trypsinized and propagated for two passages under G418 selection. Passage 6 G418 resistant cells were obtained and used for inductive expression. When they were 80% confluent, the cell cultural medium was replaced by inductive medium (DMEM/F12+10 ng/mL EGF +1% ITS +5 mg/mL prolactin +1 mg/mL hydrocortisone). Total RNA was isolated using RNA easy kit (Qiagen, USA), reverse-transcribed (Invitrogen, USA) and PCR amplified *hLF* with the *GAPDH* mRNA as a reference gene. Non-transgenic MECs were used as a negative control. The supernatants after induction were concentrated and subjected to Western blot by the standard protocol. The rabbit anti-human Lactoferrin antibody (1∶1000, Abcam, USA) and the secondary antibody was donkey anti-rabbit IgG (1∶5000, LI-COR, Gemany).

### Preparation of Goat Fibroblasts

Goat fibroblasts cells (GFCs) were obtained from the ear of a 2-month-old healthy female Saanen dairy goat (Yangling, China). The ear tissue was cut into small pieces. Tissue explants were cultured in dulbecco modified eagle medium (DMEM F-12, Sigma, USA), supplemented with 10% fetal bovine serum (FBS, Gbico, USA) and 1% (v:v) penicillin/streptomycin (10000 U/mL penicillin G, 10000 mg/mL streptomycin, Sigma, USA) at 37.8°C in 35-mm tissue culture plates in a humidified atmosphere of 5% CO_2_ in air. Cultured for 10 days, the explants were removed, and cells were harvested by trypsinization, counted, and seeded in 75 cm^2^ tissue culture flasks. When the cells reached confluency, they were collected by trypsinization and frozen in DMEM-F12 supplemented with 40% FBS and 10% dimethyl sulfoxide (DMSO, Sigma, USA).

The GFCs was identified using immunofluorescence. The cells were fixed with 4% formaldehyde for 10 min, permeabilized with 0.2% Triton X-100 in PBS for 10 min, and unspecific binding was blocked with a 2% solution of bovine serum albumin (BSA) in PBS for 1 h. The cells were then incubated with anti-Vimentin (Santa Cruz, USA) overnight at 4°C. Alexa Fluor 488-conjugated antibody was raised in goat and directed against immunoglobulins of the species used to raise the primary antibody, were used for detection of the bound primary antibodies (1∶200, 1 h). Between each step the cells were rinsed with PBS. For a negative control, the entire procedure was followed except that the primary antibody was replaced with PBS. A light-scanning microscope was used to examine the cells.

### Transfection, G418 and EGFP Selection of GFCs

Passage 4 fibroblast cells were transfected with the purified linearized vector pBC1-hLF-Neo-IRES2-EGFP with pvuI enzyme using the Lipofectamine LTX™ (Invitrogen, USA), following the manufacturer’s guidelines. Briefly, 2×10^5^ cells at passage 4 were seeded in a 35-mm culture plate one day before transfection. Cells were transfected with 4 µg of DNA. After transfection, the cells were exposed to 1000 mg/ml of G418 (Gibco, USA) for 15 days. After that, 500 mg/ml of G418 was used during the selection process to obtain stable expression, and single colonies were isolated in the presence of G418 and expanded. Single transfected cell colonies were picked up by using cloning discs (Fisher Scientific, Springfield, Nanjing, China) and placed in 24-well plates. Cells in each well were examined for EGFP expression under fluorescent light using a standard fluorescein isothiocyanate (FITC) filter setting and marked as positive and negative cell lines. When confluence was achieved, cells were passaged to 6-well plates and then to 35-mm plates. A portion of the cells from each EGFP-positive cell lines were plated for DNA isolation, quantity of EGFP expression, and cell cycle distribution. The remaining cells were frozen in DMEM-F12 supplemented with 40% FBS and 10% (v:v) DMSO.

### Identification of Transfected Cells by PCR and Cell Cycle Distribution

The G418-resistant and EGFP positive cell clones were analyzed by PCR to confirm transgene integration. Genomic DNA was isolated from each cell line using a Genomic DNA kit (Tiangen, China). PCR was used to identify the presence of *hLF* gene following procedure of pre-denaturing at 94°C for 5 min, and 30 cycles with denaturing for 30 s at 94°C, annealing for 30 s at 62°C, extension for 45 s at 72°C, and a final extension for 5 min at 72°C. To avoid non-specific amplification, the PCR primers were designed to amplify the 750 bp product covering part (1-342 bp) of the vector sequence and part (343-750 bp) of the *hLF* gene. The forward and reverse primer was 5′-GAATGGCTGGCAGTGAAACA-3′ and 5′-CTCAATGGGCTCAGGTGGAC-3′, respectively.

Cell cycle and EGFP expression analysis of transfected cells after PCR were analyzed by flow cytometry (FACS-CALIBUR, Becton Dickinson, Sunnyvale, CA). The cells were cultured in 6-well plates and grown in DMEM containing 10% FBS until 100% confluent. Then the cells were cultured for additional 2 days into contact inhibition. They were harvested using 0.25% trypsine-EDTA (Sigma, China) and re-suspended in DMEM at 1×10^7^ cells/tube. The cells were re-suspended in PBS and centrifuged at 1,500 rpm at 4°C for 5 min. The supernatant was decanted, and the cells were gently re-suspended in PBS. Cells were fixed by adding 0.7 mL cold ethanol (70%) drop-wise in a tube containing 0.3 mL of cell suspension in PBS while gently vortexing. Fixed cells were left undisturbed at 4°C for 48 h before further analysis. The fixed cells were centrifuged at 1,500 rpm at 4°C for 5 min, washed once with cold PBS, and re-centrifuged. For expression analysis of the EGFP transgene, centrifuged cells were re-suspended in 0.25 mL PBS and analyzed by flow cytometry with the instrument set for fluorescein detection. For cell cycle analysis, centrifuged cells were re-suspended in 0.25 mL PBS containing 5 µL of 10 mg/mL RNase (Sigma, USA) and incubated at 37°C for 1 h. Incubated cells were stained by adding 10 µL of 1 mg/mL propidiumiodide (Sigma, USA). The cells were then analyzed by FACS-CALIBUR at 488 nm. Non-transfected cells were used as a negative control for fluorescein and cell cycle detection. Histogram and dot plots were created using Cell Quest software (Beckton Dickinson, San Jose, CA). The percentages of cells cycle within the various phases of the cell cycle were calculated using the modfit program.

### Nuclear and Embryo Transfer

Transfected cells, which were positive for PCR amplification for *hLF*, high EGFP stable expression, normal cell cycle distribution, were used as donor cells. Oocyte collection and enucleation, nuclear transfer, oocyte activation, embryo culture, and embryo transfer were carried out with protocols as described previously [15]. First, goat ovaries were collected from an abattoir (Er’Ling Goat Abattoir, Danyang, China) with the permission of using these ovaries for normal scientific research. Cumulus-oocyte complexes (COCs) were sorted under stereomicroscopy, and cultured for 20 to 23 h at 38.5°C under 5% CO_2_ in humidified air. Matured oocytes were then pipetted with 0.3% hyaluronidase to remove granulosa cells. Those that had released their polar bodies were selected. Second, oocytes (15-20/group) were treated for 10 min in TCM199 with 7.5 g/mL cytochalasin B, 10 g/mL Hoechst 33342, and 10% FBS, and examined under fluorescence microscopy. Each oocyte was aspirated using a holding pipette. Only completely enucleated oocytes were used for NT. Third, a micropipette containing the donor cell was introduced through a slit made on the zona pellucida during enucleation. The cell was inserted between the zona and cytoplast membrane to facilitate close membrane contact and subsequent fusion. Reconstructed embryos were washed with fusion medium (0.25 M d-sorbitol, 0.5 mM (CH_3_COO)_2_Mg•4H_2_O, 0.1 mM (CH_3_COO)_2_Ca, 0.5 mM HEPES, and 1 mg/mL BSA), and then moved to microslides (BTX, Inc., San Diego, CA, USA) with fusion medium. Fusion was induced with two 1.2 kV/cm DC pulses lasting for 20 s. The fusion results were examined after 0.5 h. The embryos that were not fused were fused again. Finally, reconstructed embryos were treated with TCM199 containing 5 µM ionomycin for 5 min, incubated for 4 h in TCM 199 containing 2 mM 6-DMAP, washed twice, transferred to SOFaa, and cultured in CO_2_ incubator under 38.5°C, 5% CO_2_, and saturated humidity. At 38 to 40 h after fusion, *hLF*-gene NT embryos were implanted surgically (approximately 10 embryos per goat) into the oviducts of recipient local goats at 48 to 60 h after estrus.

### Microsatellite Analysis, PCR and Southern Blot Analysis of Cloned Goats

#### Tissue collection and genomic DNA extraction

Ear tissues were obtained from new born goats from the present study, including 3 cloned lived goats (CL1, CL2, and CL3) and 2 cloned aborted goats (CA1 and CA2, aborted at 140 days of gestation). The ear tissue of an adult recipient goat from natural reproduction was collected as a control. All samples were frozen immediately after collection and stored at −80°C until further analysis. The Institutional Animal Care and Use Committee at Nanjing Agricultural University approved all procedures involving the use of animals. Genomic DNA was extracted from the ear tissues using a Genomic DNA Kit (Tiangen, Beijing, China) for the following experiments.

#### Microsatellite analysis

The five cloned goats obtained by SCNT were subjected to parentage analysis to confirm genetic identity with the donor cells used for SCNT. DNA was extracted from the ear tissues from the goats, surrogate recipients, and donor cells. Microsatellite assay used eight caprine and bovine microsatellite markers (ETH10, ETH152, ILSTS005, ILSTS008, INRA063, INRA011, CP34, MAF65) taged with fluorescent dyes FAM [20]. All of the PCR products were run on a 3100 Genetic Analyzer. The allele sizes were determined using Genescan software (ABI, USA). Primers for the eight Microsatellite markers were listed in Table S2 in File S1.

### Genomic DNA Analyses with PCR and Southern Blot

To amplify the *hLF* gene by PCR, the nucleotide sequence of the pBC1 cloning vector (Invitrogen, USA) was used to design Identification primer I (Table S3 in File S1) corresponding to the pBC1 nucleotide sequence at positions 8,554-8,580 bp and 10,789-10,814 bp, respectively. This could yield a predicted amplification product of 2,237 bp. Each PCR reaction mixture contained 100 ng genomic DNA, 10 pmol of each primer, and 10 µL of 2×G_0_Taq Green Master Mix (Promega, Madison, Germany). The PCR procedure was pre-denaturing at 95°C for 5 min, and 30 cycles with denaturing for 30 s at 95°C, annealing for 30 s at 58.8°C, extension for 30 s at 72°C, and a final extension for 7 min at 72°C.

For Southern blot analysis, genomic DNA (20 µg) was digested with *Hind III*. The fragments containing the full sequence of the *hLF* gene were separated on the 0.7% agarose gel. For blot analysis, primers for the synthesis of probe (Table S3 in File S1) were corresponding sequence positions 8,269-8,288 bp and 9,088-9,107 bp of the vector, respectively. The probe was synthesized using a PCR DIG Probe Synthesis kit (Roche, Germany), purified by agarose gel electrophoresis, and labeled with digoxigenin before hybridization. Detection of labeled DNA on the positively charged nylon membrane was performed using a DIG luminescent detection kit (Roche, Germany).

### The Methylation Status Analysis of Genomic DNA of Transgenic Cloned Goats

#### DNA samples

Besides the DNA samples from new born goats of the present study (CL1, CL2, CL3, CA1, and CA2), an extra transgenic cloned lived goat (CL4) and an extra cloned aborted goat (CA3, aborted at 150 days of gestation) from another study were included as well. The DNA samples of three goats from natural reproduction were used as controls (NL1, NL2, and NL3).

#### Identification of putative DMRs of imprinted genes in goat genome

The DMRs of imprinted genes have regions that are highly conserved across various mammal species. Therefore, we compared the well-defined DMRs of *H19* and *IGF2R* in human, bovine, and ovine genomes to identify the counterparts of these DMRs in goat genome, using an online software MethyPrimer with restrictive conditions (GC percentage >50.0%, CpG observe/expect >0.6) to identify the distribution of CpG islands.

#### Bisulfite treatment of oligonucleotides and primers design

Bisulfite treatment and recovery of DNA samples were carried out with the EpiTect Bisulfite kit (Qiagen, USA), following the manufacturer's instructions. Briefly, 2 µg DNA in 20 µL volume was used for each reaction and mixed with 85 µL bisulfite mix and 35 µL DNA protect buffer. Bisulfite conversion was performed on a thermocycler as follows: 99°C for 5 min, 60°C for 25 min, 99°C for 5 min, 60°C for 85 min, 99°C for 5 min, 60°C for 175 min and 20°C indefinitely. The bisulfite-treated DNA was recovered by EpiTect spin column and subsequently sequenced to confirm the efficiency of bisulfite conversion.

The specific primers for amplification and sequencing of DMRs of *H19* and *IGF2R* were designed using the Primer Express software application (ABI, CA). Primers for bisulfite treated DNA were optimized to amplify a fragments of 363 bp for *H19* and a 244 bp for *IGF2R* at annealing temperature of 55-65°C. We designed the primer spanning the DMR encompassing CTCF3 site of *H19* gene (F: 5′-TATTAGGTTTTTGGTGGTATAGAG-3′, R: 5′-CTCTCCTCTCCCAACTTCAA C-3′). For *IGF2R*, we designed the primers spanning the DMR in intron 2 (F: 5′-GTTTTATGGTYGTYGGTAGAGGTA-3′, R: 5′-CRCCRAATCCTACAA ACCCTAC-3′; Y =  C/T, R =  A/G).

#### Bisulfite PCR and sequencing

Touch Down PCR for *H19* was performed with a 2×PCR master mix solution (TaKaRa, JA) as following procedures: one cycle of 95°C for 5 min; two cycles of 95°C for 30 s/60°C (annealing Tm) for 1 min/72°C for 1 min; two cycles of 95°C for 30 s/58°C for 1 min/72°C for 1 min and so forth; lastly, 25 cycles of 95°C for 30 s/50°C (annealing Tm) for 1 min/72°C for 1 min, 72°C for 7 min. The PCR procedure for *IGF2R* was the same as *H19*, but the annealing Tm ranged from 62°C to 54°C. The PCR products were recovered and purified with Wizard SV Gel and PCR Clean-Up System (Promega, USA) following the manufacturer’s protocol and subcloned into pMD19-T cloning vector (TaKaRa, JA). Positive transformants colonies (n = 15) were sequenced by Big Dye (ABI, USA) on an ABI PRISM Model 3730 and analyzed using the Sequencer software application version 4.1.4 (Gene Codes, USA). After bisulfite treatment, unmethylated cytosine residues turns into thymine residues, while methylated cytosine residues remain as cytosine residues. The methylation patterns were analyzed only using sequences derived from more than 10 clones with >99% cytosine conversions only, with BIQ Analyzer software. The methylation percentage of every sample was obtained from BIQ Analyzer software. DNA methylation among the three groups were tested by one-way ANOVA, statistical analyses were performed using GraphPad Prism (version 5.03, San Diego, USA). Differences were considered significant at P<0.05.

## Results

### Selection of Competent Donor Cells for SCNT

#### Culture and identification of stable GFC lines

**Figure 2:**
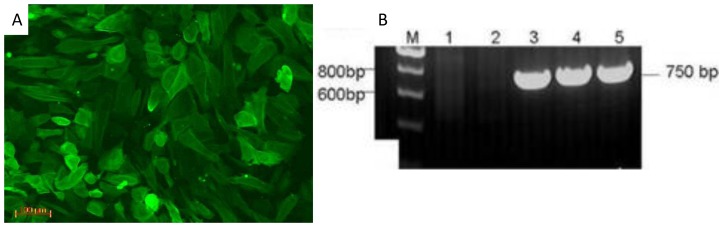
EGFP (A) and PCR identification (B) of goat fibroblast cell lines transfected by vector pBC1-hLF-Neo-IRES2-EGFP. (A) G418-resistant colony of fibroblast cells at fifth passage with EGFP fluorescence (×20). (B) M: DNA marker. Lanes 1 and 2: Non-transfected goat fibroblasts cells as negative controls, lanes 3 to 5: transgenic cells.

The GFCs were isolated and purified to be used as donor cells preparation (Figure S1). After transfection with constructed vector and G418 selection, 64 G418-resistant cell clones were obtained. Then, 34 clones expressing EGFP (Figure 2A) were selected for PCR identification and resulted in 28 positive clones (Figure 2B).

#### Cell cycle distribution after contact inhibition

Among 28 EGFP and PCR positive GFC clones, 23 were with good status and selected for the following cell cycle test. The results indicated that the average G0+G1 ratios of 19 cell clones were 83.45% (SE  = 2.96%), which was not significantly different from the non-transfected control cells (87.26%, SE  = 0.42%) (Table S3 in File S1). These results confirmed that these 19 transgenic cell clones were competent as donor cells for SCNT.

### Inductive Expression of *hLF* cDNA *in vitro*

**Figure 3:**
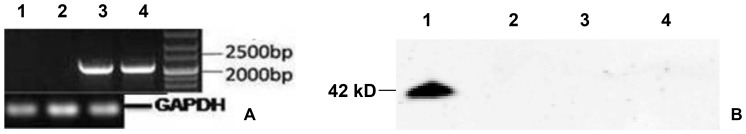
RT-PCR (A) and Western blot analysis (B) of inductive expression of hLF transgene. (A) Lane 1: non-transgenic mammary epithelial cells, lane 2: transgenic fibroblast cells, lane 3: transfected mammary epithelial cells, lane 4: vector pBC1-hLF-Neo-IRES2-EGFP as positive control. GAPDH was used as a reference gene. (B). Lane 1: Recombinant hLF (42 kDa) was detected in the supernatants from transfected mammary epithelial cells cultured in the inductive medium, lane 2: No signals were present in the supernatants from transfected fibroblasts, lane 3: No signals were present in the supernatants from transfected epithelial cells without inductive medium, lane 4: no signals were present in the supernatants from normal epithelial cells with inductive medium.

*hLF* mRNA was present in the transfected MECs under the induction of prolactin, ITS and hydrocortisone. Neither non-transgenic MECs nor transgenic fibroblast cells can express *hLF* cDNA of 2,136 bp even with a hormone signal (Figure 3A). Western blot also showed that the recombinant *hLF* was present in the supernatants of transfected MECs with the inductive medium, but not in the transfected fibroblasts and non-inductive MECs, which further confirmed mammary-specific expression of *hLF* transgene. However, the recombinant *hLF* was of a smaller mass (about 42 kDa) than standard hLF (80 kDa) (Figure 3B).

### Production of *hLF* Transgenic Cloned Goats

In total, 240 embryos were transplanted into 23 local recipient goats. Pregnancy was assessed on a continuous basis throughout gestation. Recipients were examined every morning and evening with healthy adult male goats. Recipients showing estrus performance were regarded as non-pregnancy and used as recipients for embryo transfer later. The recipients which did not show estrus until 40 days after embryo transfer were regarded as pregnant. In this study, 5 of 23 (21.7%) pregnancies were established on day 40 after embryo transfer. Three of them went full term. The birth body weight of cloned offspring was 2.70, 2.75, and 2.95 kg, respectively. Two pregnancies were lost around 140 days of gestation. The ears were collected for the following experiments. The lived cloned goats and aborted fetus were all females, which was consistent with the gender of donor cells.

### Identification of Cloned Transgenic Status for All Clones

The five cloned goats showed the same alleles for 7 of the 8 Microsatellite loci as the donor cells (Table 1), but only showed the same alleles for one locus as the recipient goat, which might be a coincidence. These results confirmed the clone status of five transgenic goats.

**Table 1:** Microsatellite analysis of receipt goat, donor cells, three lived and two dead transgenic cloned goats.

Goats	Allele of Microsatellite Markers							
	ETH10	ETH152	ILSTS005	ILSTS008	INRA063	INRA011	CP34	MAF65
Receipt goat	205/207	199/201	181/183	177/177	164/166	230/238	116/118	130/136
Donor cells	207/207	203/203	181/183	177/179	166/166	216/216	114/120	124/136
Live 1	207/207	199/203	181/183	177/179	166/166	216/216	114/120	124/136
Live2	207/207	203/203	181/183	177/179	166/166	216/216	114/120	124/136
Live3	207/207	199/199	181/183	177/179	166/166	216/216	114/120	124/136
Dead 1	207/207	203/203	181/183	177/179	166/166	216/216	114/120	124/136
Dead 2	207/207	203/203	181/183	177/179	166/166	216/216	114/120	124/136

**Figure 4:**
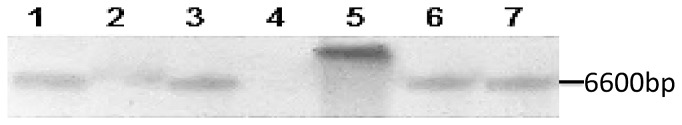
Southern blot for identifying the transgenic cloned goats. Lane 1-3: there lived goats, respectively; lane 4: recipient goat as a negative control; lane 5: vector pBC1-hLF-Neo-IRES2-EGFP as a positive control; lane 6 and 7: two dead goats, respectively. The expected size of the fragment is 6,600 bp.

Southern blot on genomic DNA (using probes specific for *hLF* and *β-casein* genes) produced fragments of the expected size for both lived and dead cloned goats (Figure 4, Figure S2). This suggested that all 5 goats were transgenic.

### DNA Methylation Status of *H19* DMR

**Figure 5:**
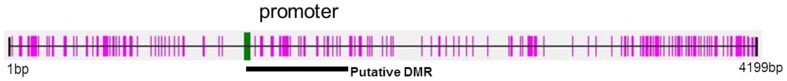
Putative DMRs of *H19.* Schematic of CpG site distributions in the putative DMRs of *H19*; Vertical red lines represent each CpG site. Horizontal gray bars represent analyzed regions and DNA length. Horizontal rectangular bar represents the putative DMR. Vertical green line represents start site of the putative DMR, located between nt 1,321 and nt 1,683 (GenBank accession no. EF577239.1).

The putative DMR of *H19* was located upstream of the promoter, between 1,321 and 1,683 nucleotides (nt) (Figure 5, GeneBank accession no. EF577239.1). This DMR showed 93% identity with that of sheep (GeneBank accession no. AJ566210). Also, this DMR contained one minimal CTCF (zinc-finger protein putative binding site) motif (CCGNNGGNGGC) [21], and regarded as CTCF III according to the position.

**Figure 6:**
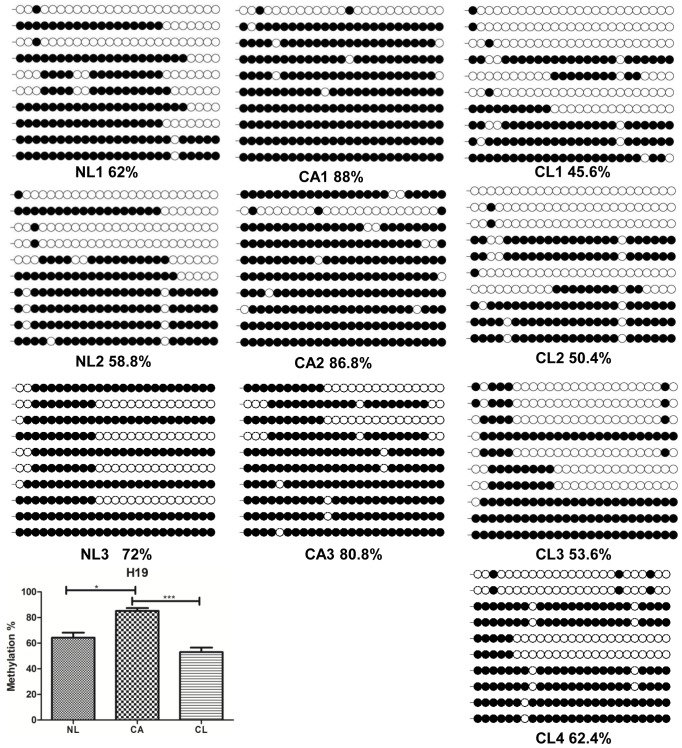
DNA methylation status of *H19* DMR. Methylation status of *H19* DMR in goats from normal reproduction (NL), cloned lived transgenic goats (CL) and cloned aborted transgenic goats (CA). Unfilled (white) and filled (black) circles represent unmethylated and methylated CpGs, respectively. Horizontal line of circles represent one single clone that was sequenced. Lollipop diagrams were generated by BIQ Analyzer software [35]. The data was analyzed by one way ANOVA.* (P<0.05) denotes significant differences between NL goats and CA goats, *** (P<0.001) denotes very significant differences between CL goats and CA goats.

As shown in Figure 6, methylation level of *H19* DMR in the cloned aborted goats (CA1-3) was significantly higher than the goats from natural reproduction (NL1-3) (P<0.05) and lived cloned goats (CL1-4) (P<0.001). There was no significant difference between NL and CL goats.

### DNA Methylation Status of *IGF2R* DMR

**Figure 7:**
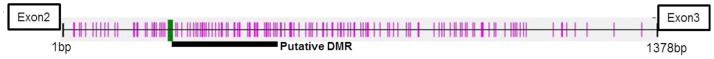
Putative DMRs of *IGF2R.* Schematic of CpG site distributions in the putative DMRs of imprinted gene *IGF2R*; Vertical red lines represent each CpG site. Horizontal gray bars represent analyzed regions and DNA length. Horizontal rectangular bar represent the putative DMR. Vertical green line represents start site of the putative DMR, located between nt 244 and nt 475 (GenBank accession no. EF577240.1).

**Figure 8:**
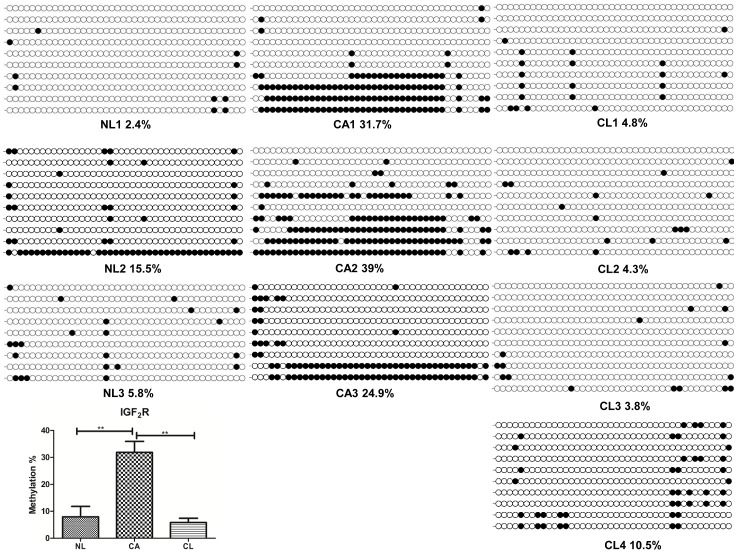
DNA methylation status of *IGF2R* DMR. Methylation status of *IGF2R* DMR in goats from normal reproduction (NL), cloned lived transgenic goats (CL) and cloned aborted transgenic goats (CA). Unfilled (white) and filled (black) circles represent unmethylated and methylated CpGs, respectively. Horizontal line of circles represent one single clone that was sequenced. Lollipopdiagrams were generated by BIQ Analyzer software [35]. The data was analyzed by one way ANOVA. **(P<0.01) denotes very significant differences between NL group and CA group, CL group and CA group, respectively.

As shown in Figure 7, the putative DMR of *IGF2R* is located between 244 and 475 nt of intro 2 (GeneBank accession no. EF577240.1). Methylation level of *IGF2R* DMR (Figure 8) in the cloned aborted goats (CA1-3) was significantly higher than the natural reproduction goats (NL1-3) (P<0.01) and lived cloned goats (CL1-4) (P<0.01). There was no significant difference between NL and CL goats.

## Discussion

In the present study, we firstly obtained 5 *hLF* transgenic cloned goats by an effective and reliable procedure. Three of the goats were birth lived, while another two died in gestation. Therefore, we further investigated the underlying epigenetic molecular mechanism of regulating the development of cloned animals by studying the DNA methylation of two imprinted genes (*H19*, *IGF2R*). Hyper-methylation of *H19* and *IGF2R* genes was observed in CA goats, while DNA methylation in CL goats was not significantly different from NL goats.

MECs have been widely used as a model for studying the regulation of milk protein gene expression [22]. So we isolated primary MECs from Saanen dairy goats at lactating phase. MECs were used to examine the activity of *hLF* expression cassette of plasmid. Consistent with previous studies on transgenic expression of MEC lines in other species [23], [24], the expression of recombinant *hLF* could be detected (Figure 3). The result confirmed that the activity of *hLF* expression cassette was effective for generation of *hLF* transgenic cloned goats. The differences in size of recombinant protein and natural protein have been widely observed. The mechanism is still not fully understood yet. In van Berkel *et al*. [25] and Peng *et al*. [26], the relative molecular mass of recombinant hLF was about 1–2 kDa smaller than that of natural hLF. This has been probably attributable to the differences in N-linked glycosylation. In the present study, the molecular weight of recombinant hLF was also smaller, which may be because the transgenic *hLF* was generated by the different patterns of post-translational modifications such as phosphorylation, glycosylation, sulfation, and ubiquitination between human and MECs.

The source of donor cells is an important factor affecting the efficiency of NT. Somatic cells have been mainly contributed to production of transgenic livestock, because embryonic stem cell (ESC) lines have not been obtained yet. Induced pluripotent stem cells (iPSC) are only limited to the studies in laboratory at the present stage [27], [28]. The fetal fibroblast cells are the main source of donor cells for SCNT. There are still some proposed disadvantages of using fetal fibroblast cells such as comprehensive sex identification, unknown cell source, animal ethnics, high aborted percentage of offspring [10], [29], [30]. Lived clones obtained from adult somatic cells in sheep [31], [32], cattle [33]-[36], and pigs [37]-[40] have also been reported. Kurome et al. indicated that there was no significant difference for the effects of different donor cell sources on cloning success [41]. In the present study, we used fibroblast cells isolated from ear tissue of the two-month-old dairy goat as donor cells for SCNT. We obtained a pregnant percentage of 21.7%, which is similar to that of using fetal fibroblast cells [42]. The possible reason is that the fibroblast cells of two-month-old goats partly share the characteristic of rapid propagation and potential differentiation of fetal fibroblast cells. Also, two-month-old goats also showed the genetic merit about health. The selection of goats for donor cells from healthy goats is more reliable than from a fetus with unknown phenotype. Furthermore, isolating fibroblast cells from two-month-old goats is easier and non-invasiveness to the animals. In our lab, seven lived transgenic cloned goat doeling has been obtained using transgenic fibroblast cells from three-month-old goats [9]. In another study in our lab using transgenic fibroblast cells from two-month-old goats, 47% pregnant percentage has been attained (unpublished data). Therefore, fibroblast cells from two-month-old goats might be an alternative to produce transgenic cloned goats by SCNT.

Previous reports showed that not all of the cells that survived after antibiotic selection were transgenic because of the bystander effect (transgenic cells with the antibiotic-resistance gene can provide protection to nearby non-transgenic cells either by secretion of the gene product into the medium or by direct cell-to-cell contact) [43]-[46]. Screen of cloned embryos for EGFP before embryo transfer can be used to improve the efficiency of transgenic animals production. However, UV light in this procedure may reduce the development and implantation percentage of transgenic cloned embryos [29]. Therefore, pre-screen of donor cells using extra markers such as EGFP [29] and confirmation procedures such as cell cycle distribution and flow cytometry analysis of EGFP expression percentage before NT to get pure transgenic cells were used in our study instead. In the present study, 34 of the 64 G418-resistant cell clones expressed EGFP. After PCR, cell cycle and flow cytometry analysis, only 19 cell clones were qualified as donor cells for SCNT. Therefore, the procedure of sequential selection of transgenic cells lines may improve the efficiency of the production of transgenic animals.

The birth body weights of the 3 lived transgenic cloned goats (2.70, 2.75, and 2.95 kg, respectively) were similar to that of normal dairy goats (3.1 kg). One of the CL goats died from Coccidiosis around 3 months of age. For the two CA goats, no apparent veterinary abnormality of development in organs was found. Recently, incomplete or non-appropriate epigenetic modification of transplanted nuclei in enucleated oocytes was proposed to be the primary cause of development failure of cloned animals [47]. An important epigenetic mechanism confirmed was genomic imprinting which plays an important role in the reprogramming of transplanted nuclei in the enucleated oocytes [48]. The majority of imprinted genes have roles in fetal growth and development. Both the maternal and paternal genomes are required for normal development [49]. *H19*, *IGF2* and its receptor (*IGF2R*) are three frequently studied imprinted genes regulating early fetal growth, and are essential for normal development of embryos [50].

The methylation status of *H19* DMR shows a semi-methylation pattern in mouse and human study [50]. Studies in mouse have demonstrated that the *H19* DMR regulates the expression of both *H19* and IGF2 [51]. In ovine, the CpG island surrounding the CTCF III binding motif *H19* DMR is subjected to differential methylation [52]. In this study, we found four putative CTCF sites of goat *H19* using CCGNNGGNGGC motif. The number is the same as the ovine [21]. The methylation status of this DMR in the CA goats was significantly higher than the NL and CL goats. There was no significant difference between NL and CL goats (Figure 6). This was consistent with the findings in cloned mice [16], cattle [53], and pigs [12], [17].

Comparative analysis of the *IGF2R* sequence indicated that the putative DMRs was located in intron 2, which is consistent with the finding in mouse, bovine, and human [47]. Missing of *IGF2R* imprinting is related with large offspring syndrome in sheep [54]. In the present study, the methylation pattern of *IGF2R* DMR in the NL goats and CL goats showed similar methylated pattern. However, the CA goats showed significant hyper-methylation (Figure 8). This is consistent with the findings in the cloned bovine [53], [55] and sheep [52], but different from the findings of Su et al. [53], who showed the *IGF2R* in dead cloned bovine was significantly hypo-methylated compared with controls.

Combining the biological functions of *H19* and *IGF2R* [12], the results of methylation status indicated that aberrant methylation of *H19* and *IGF2R* in CA goats may lead to the biallelic or large variable expression of genes, then may affect the cloned embryos reprogramming, finally affect the normal development of embryo and fetus. On the other hand, the normal methylation of imprinting genes in CL goats, which allow their appropriate expression as in NL goats may indicate the normal development of cloned embryo and fetus in the gestation, which could explain the survival of CL goats. This also confirmed the importance of these two imprinted gens in the development of the fetus. Thus, the methylation status of *H19* and *IGF2R* DMRs might be considered potentially to assess the reprogramming status of transgenic cloned animals.

In conclusion, our studies indicated that SCNT can be used to generate *hLF* transgenic goats. We got 3 lived transgenic goats and 2 dead during gestation. To the best of our knowledge, this is the first time that methylation pattern of imprinted genes in lived and dead transgenic cloned goats were described. We confirmed that DNA methylation of *H19* and *IGF2R* imprinted genes in the lived transgenic cloned goats was more normal than that of dead ones. These results will help further understanding of the importance of imprinted genes during fetus development. These two imprinted genes might be considered potentially to assess the reprogramming status of transgenic cloned animals. This will contribute to simplify and improve the efficiency of SCNT.

## Supporting Information

Figure S1 Primary goat fibroblast cells culture and immunofluorescence for the cell marker. (A) Primary goat fibroblast cells (magnification×50). (B) purified goat fibroblast cells (magnification×100). (C) left panel, positive staining of Vimentin, as the maker of fibroblast cells; middle panel, nucleus stained with Hochest 3342; right panel, merged image.
(TIF)

Figure S2 Full scans of original southern blot for data in Figure 4.
(TIF)

File S1 Supporting tables. Table S1, Primers for the plasmid construction and PCR identification for transgenic cloned goats. Table S2, Primers for the Microsatellite loci markers, there are total 8 pairs of primers modified by6-FAM for Microsatellite loci markers. Table S3, Cell-cycle distribution percentage in fluorescence and PCR positive donor cell clone lines after contact inhibition.
(DOCX)
