# Supplementary figures and images for: Generation of Five Human Lactoferrin Transgenic Cloned Goats Using Fibroblast Cells and Their Methylation Status of Putative Differential Methylation Regions of IGF2R and H19 Imprinted Genes
Source: PLoS One. 2013 Oct 30;8(10):e77798. doi: 10.1371/journal.pone.0077798 (PMC3813735; doi:10.1371/journal.pone.0077798)

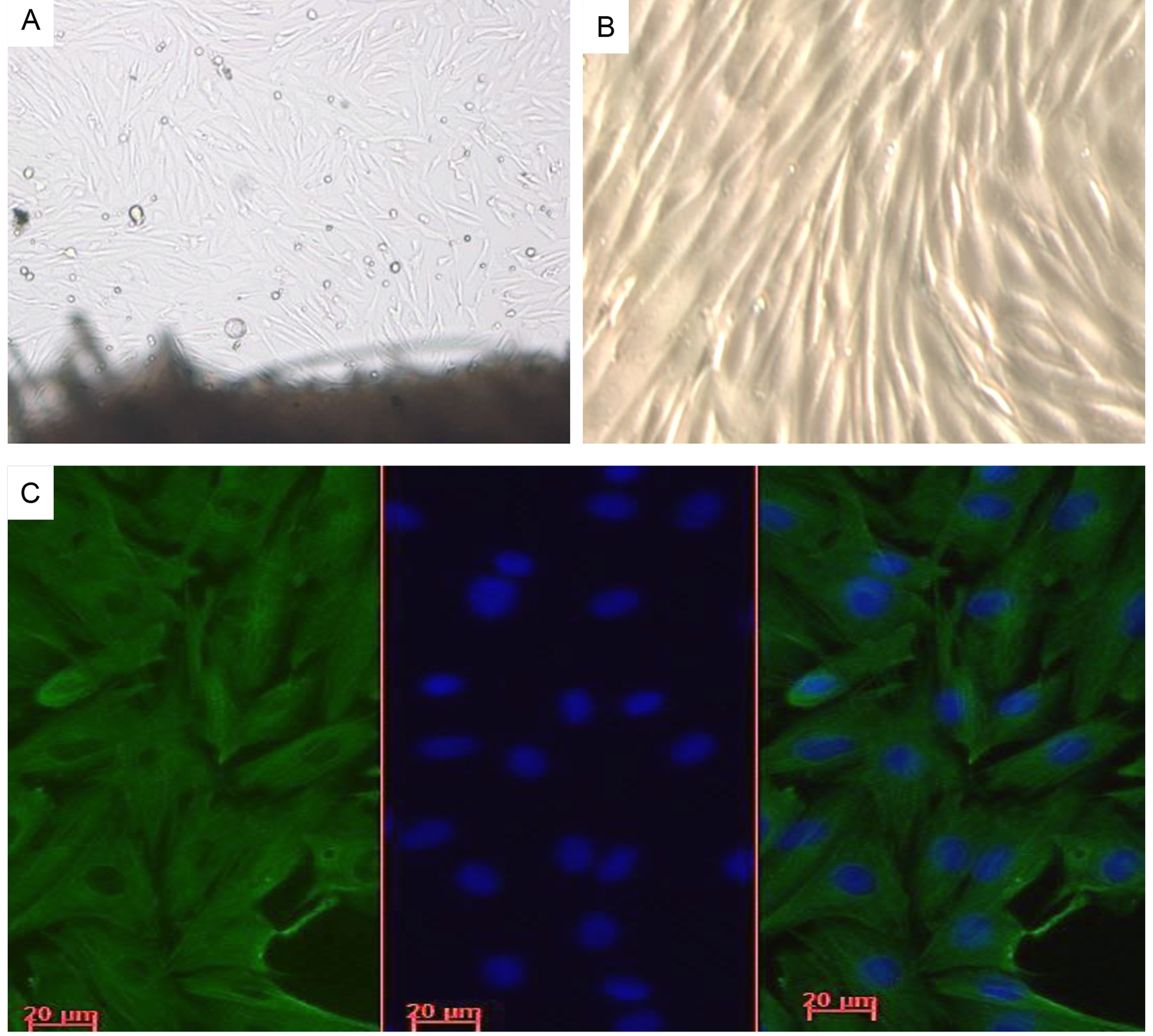

Supplement: Figure S1 — Primary goat fibroblast cells culture and immunofluorescence for the cell marker. (A) Primary goat fibroblast cells (magnification×50). (B) purified goat fibroblast cells (magnification×100). (C) left panel, positive staining of Vimentin, as the maker of fibroblast cells; middle panel, nucleus stained with Hochest 3342; right panel, merged image. (TIF) [file pone.0077798.s001.tif]

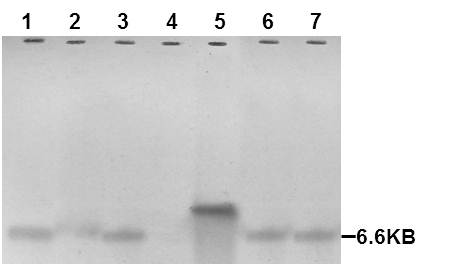

Supplement: Figure S2 — Full scans of original southern blot for data in Figure 4. (TIF) [file pone.0077798.s002.tif]
