# Supplementary material for: Generation of Five Human Lactoferrin Transgenic Cloned Goats Using Fibroblast Cells and Their Methylation Status of Putative Differential Methylation Regions of IGF2R and H19 Imprinted Genes
Source: PLoS One. 2013 Oct 30;8(10):e77798. doi: 10.1371/journal.pone.0077798 (PMC3813735; doi:10.1371/journal.pone.0077798)
Supplement: File S1 — Supporting tables. Table S1, Primers for the plasmid construction and PCR identification for transgenic cloned goats. Table S2, Primers for the Microsatellite loci markers, there are total 8 pairs of primers modified by6-FAM for Microsatellite loci markers. Table S3, Cell-cycle distribution percentage in fluorescence and PCR positive donor cell clone lines after contact inhibition. (DOCX) [file pone.0077798.s003.docx]

| Table S1. Primers for the plasmid construction and PCR identification for transgenic cloned goats   \| Primers \| Sequence of primers (5’-3’) \| Length (bp) \| \| --- \| --- \| --- \| \| Neo \| F-TCCCCGCGGAGACAGGATGAGGATCGTTTCG \| 23 \| \| R-CGCGGATCCATCTCGTGATGGCAGGTTGG \| 23 \| \| Neo-IRES_2_-EGFP \| F-GGCGGGTCGACTAGTTATTAATAGTAATCAATTACGG \| 21 \| \| R-GTAATGTCGACTTGGACAAACCACAACTAGAATG \| 23 \| \| pBC1-hLF \| F-GGGGACTGGGCAAGAGAAACTGAC \| 23 \| \| R-GCCCACCGCACACCACACG \| 20 \| \| pBC1- Neo-IRES_2_-EGFP \| F-CTTTGCCGGACAGGAGCGTAATG \| 20 \| \| R-AAACCACAGAAATGCTTGGAAG \| 22 \| \| Probe Primer \| F-GAATGGCTGGCAGTGAAACA \| 23 \| \| R-CTCAATGGGCTCAGGTGGAC \| 20 \| \| HLF \| F-CCGCTCGAGATGAAACTTGTCTTCCTCGTCCT \| 24 \| \| R-CCGCTCGAGTTACTTCCTGAGGAATTCACAGG \| 24 \| \| GAPDH \| F-GATTGTCAGCAATGCCTCCT \| 24 \| \| R-AAGCAGGGATGATGTTCTGG \| 25 \| \| Identification1 \| GATTGACAAGTAATACGCTGTTTCCTC \| 25 \| \| R-CATCAGAAGTTAAACAGCACAGTTAG \| 25 \| |
| --- | --- | --- | --- | --- | --- | --- | --- | --- | --- | --- | --- | --- | --- | --- | --- | --- | --- | --- | --- | --- | --- | --- | --- | --- | --- | --- | --- | --- | --- | --- | --- | --- | --- | --- | --- | --- | --- | --- | --- | --- | --- | --- | --- |

Table S2. Primers for the Microsatellite loci markers, there are total 8 pairs of primers modified by6-FAM for Microsatellite loci markers

| Microsatellite loci | Sequence of primers (5’-3’) | Length (bp) |
| --- | --- | --- |
| ETH10 | F-GTTCAGGACTGGCCCTGCTAACA | 23 |
|  | R-CCTCCAGCCCACTTTCTCTTCTC | 23 |
| ETH152 | F-TACTCGTAGGGAGGCTGCCTG | 21 |
|  | R-GAGACCTCAGGGTTGGTGATCAG | 23 |
| ILSTS005 | F-GGAAGCAATTGAAATCTATAGCC | 23 |
|  | R-TGTTCTGTGAGTTTGTAAGC | 20 |
| ILSTS008 | F-GAATCATGGATTTTCTGGGG | 20 |
|  | R-TAGCAGTGAGTGAGGTTGGC | 20 |
| INRA063 | F-ATTTGCACAA GCTAA ATCTAACC | 23 |
|  | R-AAACCACAGAAATGCTTGGA AG | 22 |
| INRA011 | F-CGAGTTTCTTTCCTCGTGGTGGC | 24 |
|  | R-GCTCGGCACATCTTCCTTAGCAAC | 24 |
| CP34 | F-GCTGAACAATGTGATATGTTCAGG | 24 |
|  | R-GGGACAATACTGTCTTAGATGCTGC | 25 |
| MAF65 | F-AAAGGCCAGAGTATGCAATTAGGAG | 25 |
|  | R-CCACTCCTCCTGAGAATATAACATG | 25 |

Table S3. Cell-cycle distribution percentage in fluorescence and PCR positive donor cell clone lines after contact inhibition

| cell clone (NO.) | G0/G1(%) | S(%) | G2/M(%) |
| --- | --- | --- | --- |
| 1 | 64.16 | 32.39 | 3.45 |
| 2 | 67.89 | 28.27 | 3.84 |
| 3 | 87.54 | 8.81 | 3.65 |
| 4 | 87.99 | 9.05 | 2.96 |
| 5 | 86.86 | 9.64 | 3.51 |
| 6 | 84.83 | 13.48 | 1.69 |
| 7 | 87.28 | 11.00 | 1.72 |
| 8 | 85.47 | 12.84 | 1.69 |
| 9 | 93.91 | 2.37 | 3.73 |
| 10 | 93.39 | 3.18 | 3.43 |
| 11 | 87.68 | 5.94 | 6.38 |
| 12 | 88.21 | 9.32 | 2.47 |
| 13 | 86.43 | 7.86 | 5.71 |
| 14 | 84.27 | 12.17 | 3.56 |
| 15 | 87.45 | 9.72 | 2.83 |
| 16 | 85.79 | 9.14 | 5.07 |
| 17 | 91.23 | 4.85 | 3.92 |
| 18 | 92.61 | 3.56 | 3.83 |
| 19 | 84.39 | 10.65 | 4.96 |
| 20 | 87.83 | 8.26 | 3.91 |
| 21 | 87.02 | 9.45 | 3.53 |
| 22 | 72.54 | 19.87 | 7.59 |
| 23 | 69.98 | 21.23 | 8.79 |
| Control^1^ 1 | 87.61 | 9.04 | 3.35 |
| Control 2 | 87.38 | 8.53 | 4.09 |
| Control 3 | 86.79 | 10.08 | 3.13 |

^1^Control 1-3, non-transfected cells at the 8 passage.
